# Supplementary material for: The LIM protein complex establishes a retinal circuitry of visual adaptation by regulating Pax6 α-enhancer activity
Source: eLife. 2017 Jan 31;6:e21303. doi: 10.7554/eLife.21303 (PMC5308899; doi:10.7554/eLife.21303)
Supplement: Figure 3—source data 1. — DOI: http://dx.doi.org/10.7554/eLife.21303.009 [file elife-21303-fig3-data1.docx]

*Kim et al._Fig3_source data 1*

Figure 3 – source data 1. Protein-protein interaction between LIM proteins

|  | **Lhx3** | **Isl1** | **Pax6** | **Tgfb1i1** | **Lmo4** |
| --- | --- | --- | --- | --- | --- |
| **Lhx3** |  | **+** | **-** | **++** | **++** |
| **Isl1** | **+** |  | **+** | **+** | **++** |
| **Pax6** | **-** | **+** |  | **-** | **++** |
| **Tgfb1i1** | **++** | **+** | **-** |  | **++** |
| **Lmo4** | **++** | **++** | **++** | **++** |  |
